# Use of in-silico assays to characterize the ADMET profile and identify potential therapeutic targets of fusarochromanone, a novel anti-cancer agent

**DOI:** 10.1186/s40203-015-0010-5

**Published:** 2015-06-04

**Authors:** Madison Wynne El-Saadi, Tara Williams-Hart, Brian A Salvatore, Elahe Mahdavian

**Affiliations:** Department of Chemistry and Physics, LSU-Shreveport, One University Place, 71115 Shreveport, LA USA; Department of Biological Science, LSU-Shreveport, 71115 Shreveport, LA USA

**Keywords:** ADMET, *In-silico* fishing, Apoptosis pathway, Molecular docking, Drug design, Drug optimization, Pharmacokinetic

## Abstract

**Purpose:**

For 30 years nature has provided a plethora of natural products with potential meaningful anti-cancer activity. Fusarochromanone (FC101a) is a small molecule fungal metabolite exhibiting potent *in-vitro* growth inhibitory effects and is capable of inducing apoptosis, suppressing angiogenesis and tumorigenesis, and inhibiting endothelial cell growth in multiple cancer cell lines. Despite all we know regarding FC101a, the mechanism of action and molecular target(s) of this compound have remained an enigma. Furthermore, modest *in-vivo* activity has been documented and requires addressing.

**Method:**

Early stage pharmacokinetics (PK) assessment is vital to successful drug development. Herein, we aimed to use *in-silico* assays to *i)* characterize an in-depth ADMET profile of FC101a and *ii)* to probe for possible therapeutic targets. Two-dimensional SDF files of FC101a and 13 analogs were introduced into ADMET Predictor Version 7.1 that parses the structures in order to calculate molecular descriptors, which are used to estimate ADMET properties. Calculated ADMET values were analyzed and subjected to multiple drug-like indices, delivering a PK profile of each analog. To probe for possible targets, a total of 49 proteins were introduced into SYBYL-X Version 2.0 platform and the deepest binding pocket of each protein was virtually docked with parent compound, FC101a; with the negative control, FC101b; and with the model compound, kynurenine.

**Results:**

Each analog showed promising ADMET qualities, although FC101 Oxazole was identified as the most optimized analog. Despite FC101a having a desirable ADME and toxicity profile, areas of concern were identified and must be addressed *in-vitro*. These include potential mutagenic properties and estrogen receptor toxicity. We provide potential avenues medicinal chemists could use to achieve higher effective permeation, higher blood brain barrier (BBB) penetration, and higher aqueous solubility in FC101a. Molecular docking assays revealed procaspase-8 - cFLIP(L) complex as a potential biological target and led to proposed mechanisms of action by which FC101a facilitates procaspase-8 heterodimerization, thereby increasing proteolytic activity and up regulating extrinsic apoptosis.

**Conclusion:**

Our data revealed both potential mechanisms of action and a promising ADMET profile of FC101a. These attributes render FC101a a promising lead candidate for development into a low toxic anti-cancer agent effective against a broad range of cancers.

**Electronic supplementary material:**

The online version of this article (doi:10.1186/s40203-015-0010-5) contains supplementary material, which is available to authorized users.

## Background

For 30 years nature has provided scientists a plethora of natural products with potential meaningful anti-cancer activity. Since 1940, approximately 175 small molecules have been approved as anti-cancer agents; of these, 48.6 % were a natural product or derivative (Newman and Cragg 2012). Fusarochromanone (FC101a) is a small molecule fungal metabolite exhibiting potent *in-vitro* growth inhibitory effects in 35 of 58 human cancer lines through a novel mode of action (Mahdavian et al. 2014a). FC101a demonstrates a multi-focal approach to inhibiting cancer growth that includes induction of apoptosis, suppression of angiogenesis and tumorigenesis, and direct inhibition of endothelial cell growth, evident by MTT cell viability assays, FACS analysis, and western blotting (Mahdavian et al. 2014a). Due to these characteristics, we believe FC101a is a promising lead candidate for an anti-cancer agent effective against a broad range of cancers.

Although FC101a anti-cancer activity has been well documented, its biological target(s) and mechanism of action have remained enigmatic; according to a NCI COMPARE study, its mode of action is novel when compared to a database including 50,000 compounds (Mahdavian et al. 2014a). Furthermore, diminished *in-vivo* potency remains an obstacle and requires addressing- the large disparity between levels of activity *in-vivo* and *in-vitro* suggests the molecule requires structural modifications to maximize its activity. Two questions become clear: how is *in-vivo* potency diminished, and how can we improve *in-vivo* potency? The use of *in-silico* assays is key to answering both questions and to uncovering the biological target(s) of FC101a. Although FC101a ADMET properties exclusive to Lipinski’s Rule of 5 (RO5) have been recorded, a more robust ADMET profile was unavailable until now.

**Fig. 1:**
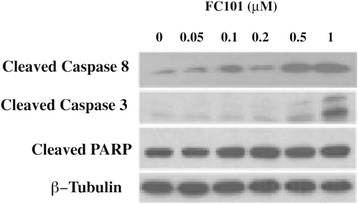
Western blot with antibodies for caspase proteins involved in apoptosis. MDA-MB-231 cells, grown in 6-well plates, were treated with FC101 (0–1 μM) for 24 h, followed by western blotting with antibodies to cleaved caspase-3, cleaved caspase 8, and cleaved PARP. Upregulation of CC8, CC3, and cleaved PARP indicate caspase activation and apoptosis upregulation in a concentration-dependent manner. β-Tubulin was used as house keeping protein

**Fig. 2:**
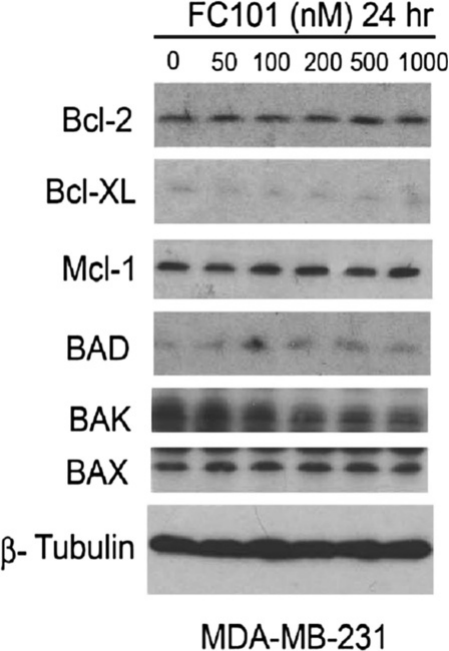
Western blot with antibodies for pro and anti apoptotic factors. MDA-MB-231 cells, grown in 6-well plates, were treated with FC101 (0–1 μM) for 24 h, followed by western blotting with antibodies to anti-apoptotic proteins (Bcl-2, Bcl-XL, Mcl-1) and pro-apoptotic proteins (BAD, BAK, BAX). Protein expression remains unchanged. β-Tubulin was used as house keeping protein

The four objectives of this study were: (1) to select optimized FC101a analogs for synthesis, (2) to characterize an in-depth ADMET profile of FC101a, (3) to discover potential avenues medicinal chemists could use to optimize FC101a, and (4) to search for the therapeutic target(s) of FC101a. Herein, we use multiple state-of-the-art computer modeling software programs and open-source platforms to perform *in-silico* assays on FC101a and multiple lead analogs; thereby generating an in-depth PK profile of FC101a and structural analogs (SA). *In-silico* fishing techniques, which require a pool of potential targets, were applied to search for the unknown biological target(s) of FC101a. Potential targets were selected for molecular docking based on previously performed cell viability and western blot assays, and availability of unambiguous crystal structures. FC101a increases caspase-3 and PARP cleavage, as well as caspase-8 activity (Fig. 1). Furthermore, Fig. 2 illustrates that FC101a does not affect anti-apoptotic proteins (Bcl-2, Bcl-XL, Mcl-1) or pro-apoptotic proteins (BAD, BAK, BAX); further attesting that FC101a induces apoptotic cascades by means of an extrinsic mechanism involving caspase-8 (Mahdavian et al. 2014a). Therefore proteins upstream of caspase-3, caspase-8, and PARP were selected as potential targets, but only if unambiguous crystal structures were available through RCSB- in total 49 proteins were selected.

## Methods

### ADMET predictor calculations

ADMET values for FC101a, 12 structural analogs, and one model compound were predicted using Simulations-Plus ADMET Predictor Version 7.1 on a Windows XP operating system. ADMET Predictor software was designed using artificial neural network ensemble (ANNE) models trained with well-defined drugs, and was chosen for it’s high prediction accuracy, and descriptor sensitivity analysis capabilities. An additional Excel file displays all predicted properties for each structure, including an out-of-scope indicator column for each model [see Additional file 1].

**Fig. 3:**
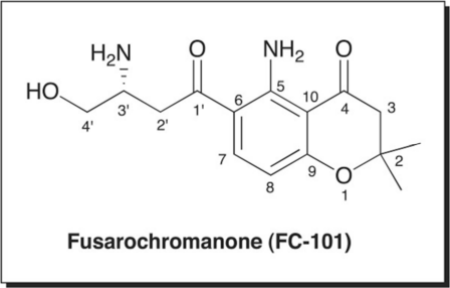
Structure of Fusarochromanone (FC101a). Two-dimensional structure of the parent compound Fusarochromanone (FC101a or FC101)

Chemical structure of FC101a (Fig. 3) and structural analogs were sketched in three-dimensional space using Tripos SYBYL-X Version 2.1 sketch mode installed on a 64GB Mac, and molecules were saved individually in MOL2 format. ADMET Predictor is unable to optimize structures; therefore energy minimization was performed in SYBYL-X prior to exporting structures into ADMET Predictor. Structures were exported into a single 2D SDF format named Jupiter04_multi21_2Dsdf.sdf and introduced into Simulations-Plus ADMET Predictor- see Additional file 2 for original .sdf file. Simulations-Plus’s Physiochemical and Biopharmaceutical Module and Toxicity Module were applied to calculate 341 molecular descriptors. Next, the software automatically uses descriptors as inputs to independent mathematical models to estimate a range of ADMET values at relevant pH 2.5 and pH 7.4. A rank ordered list of FC101a and 13 SA was generated using S + Absn_Risk and S + ADMET_Risk. Each violation contributes up to one vote “point” to the score, where points represent a liability towards oral absorption and/or toxicity in humans.

Specific ADMET values of interest included predicted logD (S + logD), predicted aqueous solubility (S + Sw), logS (value predicted by OSIRIS Data Warrior Version 4.1.1), predicted effective permeability (S + P_eff_), predicted apparent permeability (S + MDCK), and manually calculated Fsp^3^ to characterize absorption and solubility of FC101a hits. When evaluating the distribution of FC101a hits, three values were considered: percent unbound to plasma proteins (S + PrUnbnd, aka f_up_) blood-to-plasma concentration ratio (S + RBP), and volume of distribution (S + Vd). We used ADMET Predictor to both qualify and quantify the likelihood of penetrating the blood brain barrier (BBB), a requirement of all neurotherapeutic agents. A binary classifier (High/Low) and a regression model estimating the decimal logarithm of rat brain–blood partition coefficient (log [brain/blood]) were used. To determine excretion routes, FC101a hits were grouped by charge; predicted logD, molecular weight (MW), and predicted f_up,_ which were plotted in three-dimensional space and results were subjected to a known *in-silico* classification system proposed by Kusama et al. (2010). To assess for FC101a related toxicity risk, we considered predicted LD50 and TD50 values, two robust toxicity filters, and S + Pgp_Substrate model to qualify the likelihood of binding to transporter permeability-glycoprotein (P-gp). Additionally, a specificity matrix was generated to further assess the risk for potential adverse side effects due to target specificity (discussed below). Data were exported to Excel for analysis. Attention was drawn to areas of potential risk associated with oral BA and/or toxicity.

**Fig. 4:**
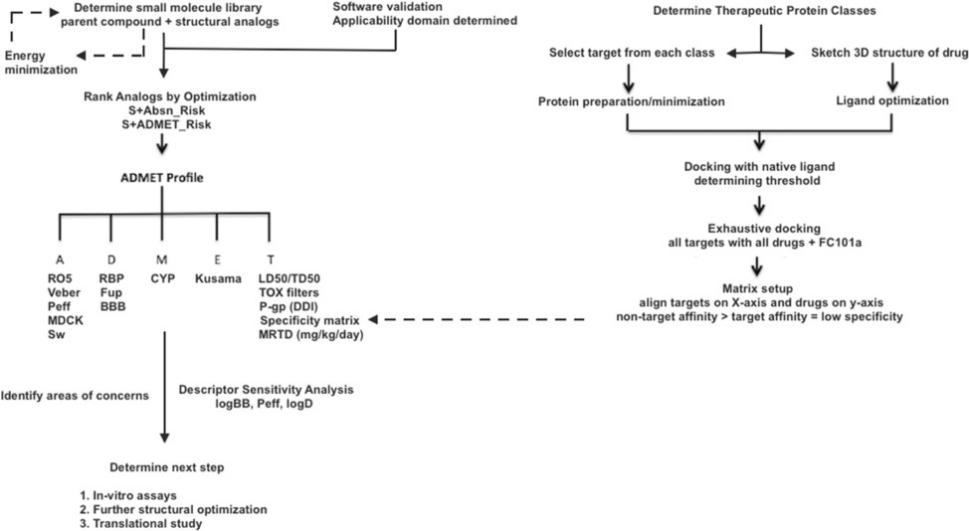
Methodology flowchart for generating ADMET profile of FC101a. Flowchart depicts the steps taken to procure predicted ADMET values and to determine the ADMET profile of FC101a

To suggest next stage optimization, relationships between certain model outputs and molecular descriptors were defined using Descriptor Sensitivity Analysis (DSA) tool. DSA determines the partial derivative of a model (i.e., S + logD, S + SW) in response to a fixed molecular descriptor. Models influencing sub-par ADMET properties of FC101a were of particular interest, and descriptors most influencing said models were examined using the DSA tool. A flowchart depicting our methodologies is illustrated in Fig. 4.

### ADMET predictor software validation

The use of ADMET prediction software has become well recognize over recent years for its role in early stage drug discovery and development. Moreover, predictive properties increase in usefulness when researchers consider the accuracy of the prediction model. In an attempt to estimate prediction accuracy, each predicted value of each drug of interest was assed to qualitatively determine if it’s calculated end-points fell within the applicability domain (AD) of the model – a default feature of Simulations-Plus ADMET Predictor. Values falling outside the AD were ignored, thus ensuring our accepted data were derived from predictive models designed with compounds occupying the same chemical space as our drugs of interest. Additional file 1 indicates out of scope values and are highlighted in magenta.

Statistical measures of the performance of linear regression models and binary classification models were checked using external test sets to further examine the overall reliability of each predictive model. Each model of interest was tested by Simulations-Plus using external test sets of drugs not included in the training process. R^2^ values between observed and predicted ADMET properties of interest, and sensitivity/specificity for binary tests were checked for models relevant to this study. Data provided by Simulations-Plus are summarized in Additional file 3, and readers should refer to his file when interested in the prediction accuracy of any model discussed within this paper.

### Determining docking protocol and active range

**Fig. 5:**
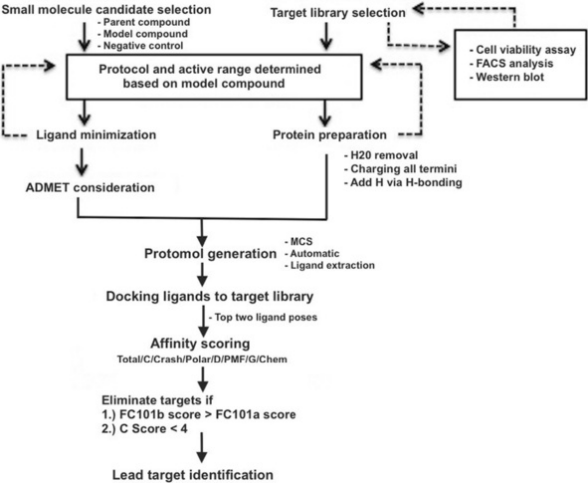
Methodology flowchart for molecular docking portion of study. Flowchart depicts the steps taken to determined a docking protocol using a model compound, kynurenine

Tripos’ SYBYL-X Version 2.1 was chosen to conduct molecular docking experiments due to it’s ability to execute multiple scoring functions at once while providing individual scores and overall consensus ranking. Molecular docking protocol and accurate consensus scoring parameters were determined from a series of dockings performed between a well-defined model compound and it’s known biological target. Kynurenine, a naturally occurring metabolite and biological substrate exhibiting structural similarity with FC101a, was selected as the model compound. Searching SciFinder structural similarity database using SMILES Notation of the following FC101a structure: C1(=C2C(=CC = C1C(CC(CO)N) = O)OC(CC2 = O)(C)C)N, returned kynurenine as the only naturally occurring metabolite (Furmanski dissertation, Unpublished Observations). The two molecules share a similarly placed o-carbonyl and aliphatic amino group along its side chain, as well as an anilinic aromatic ring. In order to *i)* optimize SYBYL-X parameters for FC101a and *ii)* determine a range of activity, multiple dockings between the model compound, kynurenine, and it’s known biological target, human kynurenine aminotransferase II (PDB IDs 2R2N and 2QLR), were first carried out. A flowchart depicting our methodology covered below is illustrated in Fig. 5.

Ten different settings were applied each time and those parameters ultimately selected as default placed kynurenine in the top binding position with a C Score ≥ 4, shown in Table 1. These parameters include GeomX setting, water molecule extraction, charging of all termini including gaps, adding hydrogens via H-bonding function, rigid protein model, and eliminating proteins with a C Score < 4. The overall structure was minimized with charges set to MMFS94 and MMfs94s force field. Potential binding sites in the protein structures were identified by generating a protomol using one of three methods; Multi-Channel Surface (MCS) mode, Automatic mode when MCS mode was unavailable, and Ligand extraction mode when interested in a specific binding pocket bound by a co-crystallized ligand. A probe radius of 1.4 Å^2^ was used and threshold was set to .50 and bloat to 1 in order to increase the size and depth of the searchable cavity for a binding site. The top two ligand poses were considered in order to account for flexibility in the binding pocket. Results from both dockings were exported to Excel for analysis and used to determine parameters for future dockings with FC101a. Although multiple scoring functions were tabulated, Total Score (default scoring function used by Surflex dock to generate and rank poses) and C Score (consensus scoring function that combines multiple scoring functions to rank the affinity of a ligand to the active site) were given priority in analysis- targets will be ranked first by C Score and then Total Score. It should be noted that Total Score is not part of the C Score consensus. This method could be a useful approach for researchers who need to generate appropriate parameters for comparable tasks, and indeed others have utilized similar approaches.

**Table 1:** Control molecular dockings between hKATII and kynurenine

Protein	Ligand	Protomol	Total Score	C Score
2R2N	pdb2r2n_ligand	Ligand extraction	8.053	5
2R2N	Kynurenine_z	Ligand extraction	6.1745	4
2R2N	FC101b_inactive	Ligand extraction	7.3148	2
2R2N	FC101a_04	Ligand extraction	6.5068	1
2QLR	pdb2r2n_ligand	Automatic mode	5.1002	5
2QLR	Kynurenine_z	Automatic mode	4.3048	5
2QLR	FC101b_inactive	Automatic mode	6.2713	4
2QLR	FC101a_04	Automatic mode	5.2248	2

These dockings were used to determine the molecular docking protocol applied to FC101a and proteins of interest. Kynurenine, represented by pdb2r2n_ligand and Kynurenine_z, are the natural substrates of 2R2N and 2QLR; all four dockings received a C Score ≥ 4, evidence to the validity of C Score in accurately ranking compounds of similar structure to kynurenine

### Ligands and protein preparation

Protein-ligand dockings were carried out on the crystal structure of 49 potential biological. Protein preparation took place prior to docking and each structure was prepared using the Atom Expression View tool and Structure Preparation tool of SYBYL-X software package. Crystal structures of proteins were retrieved from rcsb.org through SYBYL-X interface followed by preparation and staged minimization as described in the above section. Each protein was exported and saved in the following format: pdbPDBID_P.mol2, where “_P” lets the researcher know this protein has been prepared.

The structure of FC101a and both reference compounds were prepared using SYBYL-X Sketch Mode. FC101a was saved as FC101_a_04.mol2; FC101b was saved as FC101b_inactive.mol2; and kynurenine was saved as kynurenine_z.mol2. FC101b, the anti anti-cancer analog served as the negative control. This set of small molecules was saved collectively as leadset4_multi3.mol2 and underwent energy minimization using default settings.

### Molecular docking of ligands to potential FC101a targets

We created a docking setup file for each protein of interest. Protomols were generated using MCS mode and surface one was selected each time; if no channels were present, automatic mode was selected. Ligand source was set to MOL2 and leadset4_multi3.mol2 was used as the ligand source file and the job name format was: Dockingrun_pdbID_ligand source_000. Additional file 4 details the precise protocol for each protein. Upon completion, all molecules were selected and results were saved in the SYBYL-X home directory. The top two poses were selected and output format was set to spreadsheet. Data was exported as TSV format and saved in Excel for later analysis. Two circumstances resulted in the elimination of a protein from being considered: *(i)* a C Score < 4, and *(ii)* a higher affinity with the negative control, FC101b. Each docking required approximately 15 min.

### Specificity matrix generated using SYBYL-X

A specificity matrix was generated to examine FC101a specificity using targets that span the class of all known therapeutic targets, a method first performed by Shaikh et al. (2007). Eleven commercially available drugs were sketched in SYBYL-X sketch mode and saved as MOL2 files. We moved each drug into a collective file also containing FC101a- the file was named: drugmatrix_file_multi12.mol2. Molecular structures were confirmed by comparison to pubchem.org structures of the same active molecule. Crystal structures of the therapeutic target of each drug were then retrieved from rcsb.org and prepared as described in previous sections. All RCSB targets were bound to their corresponding drug; therefore, ligand extraction mode was used to extract the ligand and to generate a respective protomol. Drugs and their corresponding target, illustrated in Table 2, were selected from a data set published by Shaikh et al. (2007). We virtually docked the native ligand back into the protein to generate a threshold representing strong affinity. Each prepared protein was docked with drugmatrix_file_multi12.mol2, using MCS mode to generate protomols. Dockings were performed individually and data was saved in the manner as previously described. Drugs were aligned along the Y-axis and targets along the X-axis, ensuring row 1 (drug) matched column 1 (said drug’s therapeutic target). If drugs are specific to their target, then high affinity binding should only occur along a diagonal line. An additional file details the above protocol [see Additional file 5] and Fig. 4 provides a methodology flowchart of our full docking strategy.

**Table 2:** Approved drugs and corresponding targets used to generate 2D specificity matrix

No.	Biological target	Corresponding drug
1	Lymphocyte function-associated antigen LFA-1 (CD11A)	Lovastatin
2	Human Coagulation Factor	5-dimethyl amino 1-naphthalene sulfonic acid
3	Retinol-Binding Protein	Fenretinide
4	Human cardiac troponin C	Bepridil
5	DNA {d(CGCGAATTCGCG)}	Propamidine (TNT)
6	Progesterone receptor	Mometasone furoate
7	Platelet receptor for fibrinogen	Tirofiban
8	Human phosphodiesterase 4B	Roflumilast
9	Cyclooxygenase-2 enzyme	Indomethacin
10	Estrogen receptor	4-hydroxytamoxifen
11	ADP/ATP Translocase-1	Carboxyatractyloside

Drugs and their corresponding targets are numbered from top to bottom in same order as presented in specificity matrix. Each drug has been FDA approved and is currently on the market

## Results and discussion

### Predicting oral BA and selecting the most optimized structural analogs

**Fig. 6:**
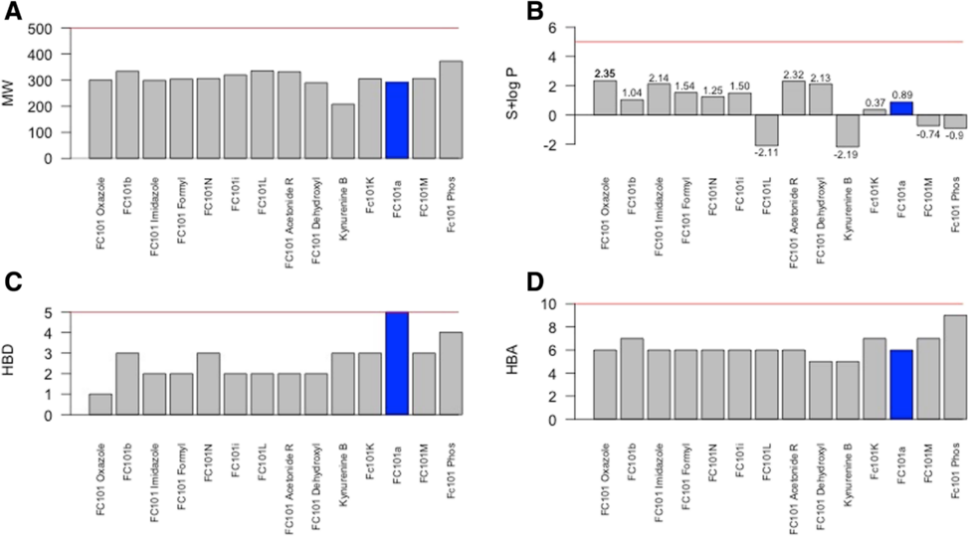
Bar graph summarizing properties of FC101a and SA in relation to Lipinski’s RO5. Bar graphs displays the distribution of properties related to Lipinski’s RO5. RO5 is perhaps the most used computational filter in drug candidate screening. Only 8.3 % of drugs within the focused WDI violated more than one of Lipinski’s rules (Lipinski et al. 1997). **a** MW, **b **S+ logP, **c **HBD, **d **HBA. All properties meet Lipinski’s RO5, denoted by the red line. FC101a is highlighted in blue

**Fig. 7:**
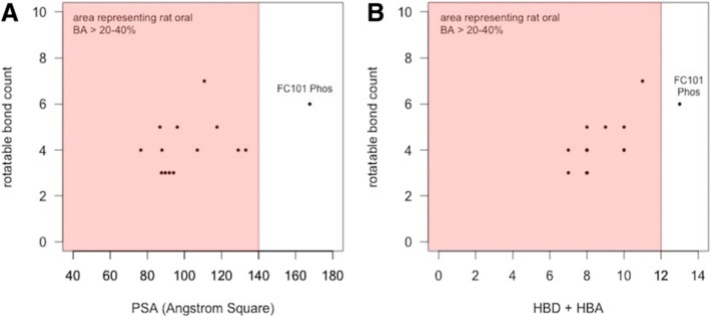
Selective criteria for drugs with rat oral BA > 20-40 %. **a** Scatter plot, rotatable bonds vs PSA, of FC101a hits. Each optimized hit is within the range of drugs demonstrating oral BA > 20-40 % in rat models- indicated by the shaded region. **b** Scatter plot, rotatable bonds VS HBD + HBA count. Regions shaded red indicate oral rat BA > 20-40 %. FC101 Phos is the only SA outside of shaded region

In order to accurately select the most optimized FC101a analogs, four drug-like indices were utilized for comparison; Lipinski’s RO5, Veber’s selective criteria for oral bioavailable drugs, and two computational filters designed by Simulations-Plus, S + Absn_Risk and S + ADMET_Risk [see Additional file 6 for chemical structure of FC101a structural analogs]. Each SA meets Lipinski’s RO5 criteria, illustrated in Fig. 6. The RO5, a guideline for identifying drugs with poor absorption and permeation, only addresses a portion of the gamut of obstacles a compound must meet to become drugable. In addition to the RO5, rules independent of MW can accurately predict oral bioavailability (Veber et al. 2002). Polar surface area (PSA) and rotatable bond count (nrot) have been found to accurately differentiate between orally active and non-orally active drugs (Ghose et al. 1999). Rat oral bioavailability data from G&G Index of 276 compounds demonstrates an “accurate and selective criteria” for rat oral BA > 20-40 % is either nrot ≤ 10 and PSA ≤ 140 Å^2^ or number of nrot ≤ 10 and HBD + HBA ≤ 12 (Veber et al. 2002). FC101a and each SA, excluding FC101 Phos, meets the before mentioned criteria as illustrated in Fig. 7. High compliance with the RO5 and rules proposed by Veber et al. (2002) suggests FC101a is likely an orally active compound in humans.

The filter S + Absn_Risk focuses on physiochemical and biopharmaceutical descriptors. Zhao et al. (2001) published human intestinal absorption data in relationship to the RO5 and Absn_Risk. Neither filter labels a drug with experimentally determined fraction absorbed greater than 50 % as “poorly absorbed.” Moreover, Absn_Risk picks 50 % more of the poorly absorbed compounds than the RO5. The latter filter, S + ADMET_Risk, serves as an all-encompassing filter including 17 weighted rules that access potential liability in relation to MW, nrot, hydrogen bond donors (HBD), hydrogen bond acceptors (HBA), PSA, overall formal charge, predicted logP (S+ logP), effective permeability (P_eff_) or apparent permeability (MDCK), aqueous solubility (Sw), percent of drug unbound to plasma (f_up_), volume of distribution (VD), toxicity, and S + Absn_Risk; thereby accumulating all ranking criteria into a single column. FC101a scored 0.99 and 1.99, respectively, resulting from high HBD charge, high steady-state volume of distribution, and potential mutagenicity (discussed later). In an effort to eliminate the poorest performing compounds, we sought to eliminate those analogs performing poorly for both filters, using a predetermined cut-off point based on scores from the poorest performing compounds in Simulations-Plus WDI. The focused WDI comprises a subset of orally bioactive drugs determined by Simulations-Plus following methods similar to Lipinski et al. (1997). FC101 Phos exceeds the cut-off for S + Absn_Risk only; therefore, we were unable to narrow our drug library (Table 3). FC101a Oxazole is the only SA scoring 0 for all computational filters and therefore considered the most optimized SA (Table 3). It is important to note druglikeness indices (i.e., S + Absn_Risk and S + ADMET_Risk) are limited in their predictive power and only estimate the druglikeness of a given compound, evident by the small portion of focused WDI compounds that achieved relatively high liability scores (10 % of WDI scored ≥ 3.5 and > 6.5 for S + Absn_Risk and S + ADMET_Risk, respectively).

**Table 3:** Ranking of FC101a structural analogs by S + Absn_Risk and S + ADMET_Risk filters

Analog	S + Absn_Risk	S + ADMET_Risk
FC101 Oxazole^a^	0	0
FC101b	0	0
FC101 Imidazole	0	0.06
FC101 Formyl	0	1
FC101N	0	1
FC101i	0	1
FC101L	1	1
FC101 Acetonide R	0	1
FC101 Dehyroxyl	0	1
Kynurenine B	1.5	1.5
FC101K	1.53	1.53
FC101a	0.99	1.99
FC101M	2.36	2.36
FC101 Phos	3.5	3.5

Compounds are ranked based on S + ADMET_Risk scores with lower scores representing less potential liability. S + Absn_Risk ≥ 3.5 for ~10 % of the focused World Drug Index (WDI), and if we accept 3.5 as a cut-off to designate compounds at risk of poor oral bioavailability, then FC101a Phos is the only SA to exceed the cut-off. S + ADMET_Risk is greater than 6.5 for ~10 % of the focused WDI, and if we accept 6.5 as a cut-off to eliminate high-risk compounds, then we’re unable to eliminate any SA from our library. ^a^Highlights FC101 Oxazole, the only compound receiving a 0 score from both filters

### Characterization of the absorption PK profile of FC101a hits

**Fig. 8:**
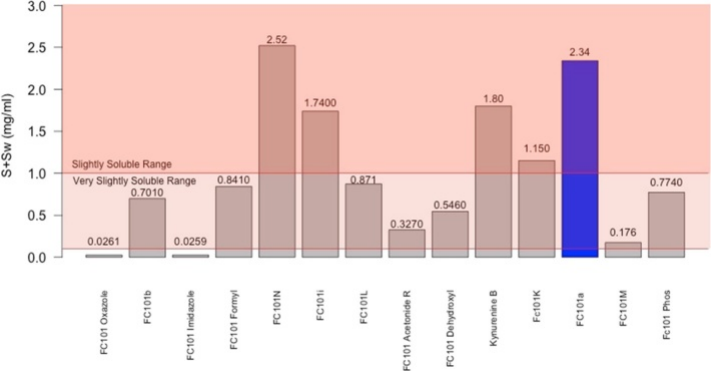
Predicted aqueous solubility of FC101a and SA. Column graph of predicted aqueous solubility of FC101a hits. All hits display solubility above Simulations-Plus’ proposed high-risk threshold of 0.01 mg/ml (not shown), suggesting the compounds display solubility similar to orally bioavailable drugs. FC101a (shaded in blue) and FC101N exhibit the highest degree of solubility and both fall within the slightly soluble range. Shaded regions indicate solubility based on United States Pharmaceutical (USP) criteria

**Fig. 9:**
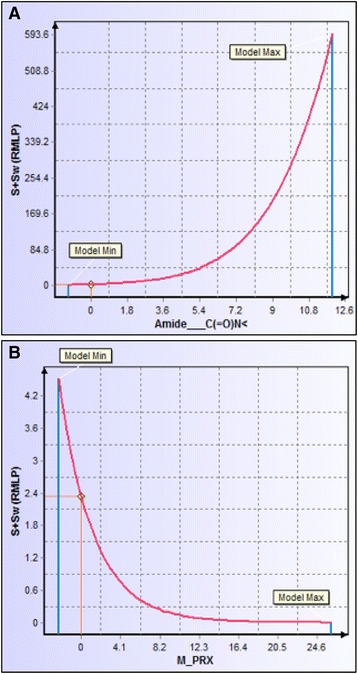
Descriptor sensitivity analysis of S+Sw to changes in most influential descriptors. **a** Sensitivity of S+SW to changes in number of amide groups. FC101a includes 0 amide groups, adding this functionality would increase aqueous solubility. **b** Sensitivity of S+Sw to the proximal effect resulting from heteroatoms nitrogen and oxygen. Lowering the proximity effect below zero would increase S+Sw

LogD provides a more meaningful barometer of lipophilicity for ionizable compounds; therefore, we used this parameter to assess the lipophilicity of FC101a, which has an ionizable 3′ amine group. Hydrophilic molecules have higher solubility, but are less equipped to readily cross the cell membrane. Hydrophilicity is represented by molecules with logD < 0, lipophilicity by molecules with logD > 0, and excessive lipophilicity by molecules with logD ≥ 3.5. FC101a and most analogs are of a modest hydrophilic nature at both pH 2.5 and pH 7.4, as shown in Table 4. All analogs avoid regions of extreme lipophilicity and extreme hydrophilicity, properties desired in orally bioavailable compounds. FC101a Oxazole and Imidazole exhibit the highest logD (2.35, 2.11 pH 7.4) and thus are likely less soluble, more permeable, and more potent than FC101a. Predicted aqueous solubility for FC101a and structural analogs are illustrated in Fig. 8. ADMET Predictor uses a soft threshold to predict solubility liability; all hits are predicted to have solubility higher than the stated minimum threshold. FC101 Oxazole and FC101 Imidazole are the least soluble analogs (S + Sw = 0.0261 mg/ml and 0.0259 mg/ml, respectively); FC101N and FC01a are the most soluble compounds (2.52 mg/ml and 2.34 mg/ml, respectively). One last means to assess solubility was manually calculating Fsp^3^. A study by Lovering et al. (2009) found increasing sp^3^ hybridized carbon saturation (as measured by Fsp^3^ = sp^3^ carbon atoms/total carbon atoms) as drugs progressed from phase I – market (0.36 for research compounds, 0.47 for marketed drugs); likewise, higher Fsp^3^ correlated with higher solubility (Lovering et al. 2009). FC101a Fsp^3^ = 0.466, the average value for approved drugs and drugs with high solubility. Our data found increasing amide groups and decreasing the proximity effect of heteroatoms nitrogen and oxygen would further increase aqueous solubility of FC101a, illustrated in Fig. 9.

**Table 4:** Comparison of logD at pH 2.5 and 7.4 for FC101a analogs

Analog	pH 2.5	Solubility (pH 2.5)	pH 7.4	Solubility (pH 7.4)
FC101 Oxazole	2.25	Lipophilic	2.35	Lipophilic
FC101b	1.04	Lipophilic	1.04	Lipophilic
FC101 Imidazole	−0.55	Hydrophilic	2.11	Lipophilic
FC101 Formyl	−1.3	Hydrophilic	0.75	Lipophilic
FC101N	−1.1	Hydrophilic	−0.53	Hydrophilic
FC101i	−1.78	Hydrophilic	0.17	Lipophilic
FC101L	−2.11	Hydrophilic	−2.11	Hydrophilic
FC101 Acetonide R	−0.66	Hydrophilic	1.67	Lipophilic
FC101 dehydroxyl	−0.91	Hydrophilic	0.06	Lipophilic
Kynurenine B	−2.27	Hydrophilic	−2.2	Hydrophilic
FC101K	−1.91	Hydrophilic	−0.03	Hydrophilic
FC101a	−1.69	Hydrophilic	−0.53	Hydrophilic
FC101M	−1.02	Hydrophilic	−0.76	Hydrophilic
FC101 Phos	−1.02	Hydrophilic	−0.9	Hydrophilic

Most analogs are hydrophilic, indicating a water-soluble nature. Some analogs like FC101 Oxazole are lipophilic and thus would more readily penetrate the lipid bilayer

**Fig. 10:**
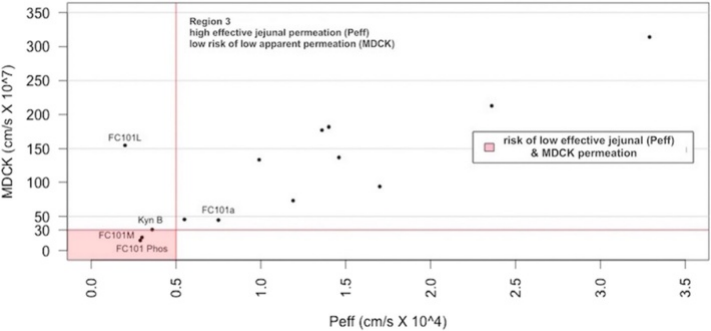
Scatter plot of predicted apparent permeability VS predicted effective permeability. Scatter plot of predicted apparent permeability (MDCK cm/s × 10^7) VS predicted effective permeability (Peff cm/s × 10^4). Region shaded in red indicates the least desirable permeability profile. Regions were determined by Simulations-Plus’ proposed high-risk thresholds, and are set at Peff < 0.5 cm/s × 10^4^ and MDCK < 30 cm/s × 10^7^

**Fig. 11:**
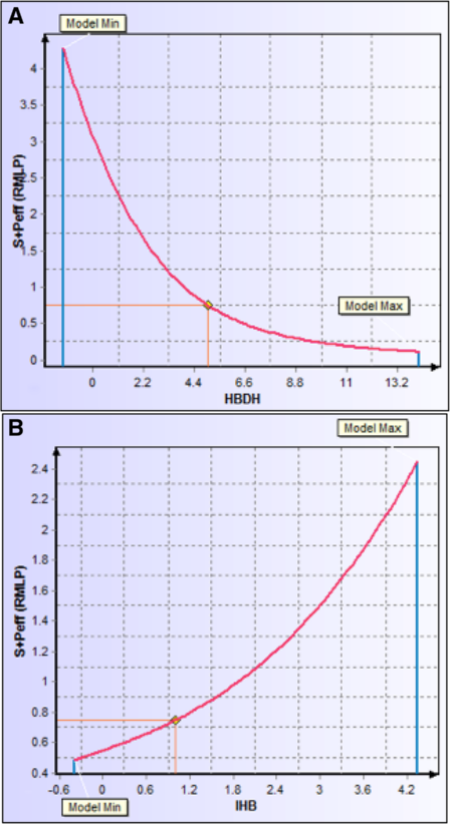
Descriptor sensitivity analysis of S + Peff to changes in most influential descriptors. **a** Sensitivity of S + Peff to number of hydrogen bond donor protons. **b** Sensitivity of S + Peff to the number of intra-molecular hydrogen bonds. Adding intra-molecular hydrogen bonds would lower the number of hydrogen bond donor protons- this method could be used to increase effective jejunal permeation

Human jejunal permeability reflects diffusion for passively transported drugs in the human jejunum- the region of the intestinal tract with the largest surface area (Lande et al. 1994; Fagerholm and Lennerniis 1995; Lennernas et al. 1997). Using a soft threshold for both apparent and effective permeability models, a four-section scatter plot was generated, evident in Fig. 10. FC101M and FC101 Phos fell within region representing low jejunal and MDCK permeation. Kynurenine B and FC101L fell within region indicating poor jejunal permeation. All other analogs, including FC101a, fell within region 2, which represents high jejunal permeation and no risk of low MDCK permeation. To further increase jejunal permeation of FC101a, Fig. 11 illustrates the effect hydrogen bond donor protons and intra-molecular hydrogen bonds have on S + P_eff_. Many researchers have documented the importance of hydrogen-bonding capacity in determining permeability of drug solutes (Waterbeemed and Gifford 2003). Decreasing hydrogen bond protons, perhaps by increasing intra-molecular hydrogen bonds would increase effective jejunal permeability. Our results are consistent with data published by Alexander et al*.* (2011) which claims intra-molecular hydrogen bonds improves membrane permeability and intestinal absorption (Alexander et al. 2011).

### Characterization of the distribution PK profile of FC101a hits

**Fig. 12:**
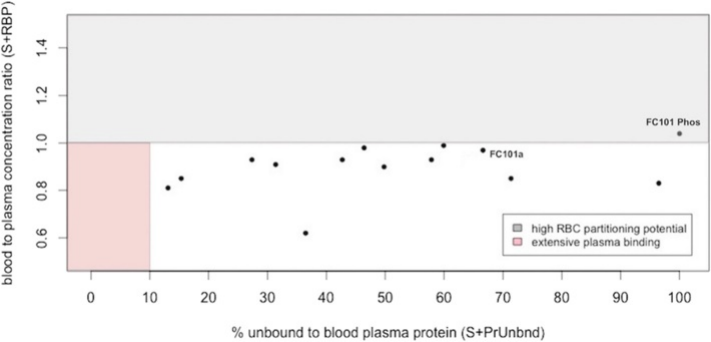
Scatter plot of predicted f_up_ VS predicted [blood-to-plasma] ratio. Scatter plot of predicted percent of drug unbound to plasma proteins VS predicted blood-to-plasma concentration ratio. The shaded regions indicate unfavorable high red blood cell partitioning and extensive plasma protein binding. For drugs with a RBP > 1.0 suggests partitioning to erythrocytes, indicated by the region shaded red. Drugs with f_up_ ≤ 10 % indicates extensive plasma binding, indicated by the region shaded gray. Industrially accepted

A percent of circulating drug may bind to plasma or whole blood proteins at various affinities, and it is widely accepted that only unbound drug may interact with intended molecular targets (Smith et al. 2001). As Fig. 12 shows, FC101a avoids regions of high red blood cell (RBC) partitioning and regions of extensive plasma protein binding, with a blood-to-plasma concentration ratio of 0.97 and 66.61 % unbound to plasma proteins. Excluding FC101 Phos, each SA avoids regions of both high RBC partitioning and extensive plasma proteins. This suggests the compounds are free of RBC metabolism and can efficiently reach the therapeutic target- an ideal characteristic of an effective drug. *In-vitro* assays should be performed to conclude our *in-silico* findings; additionally, the appropriate biological fluid must be chosen when assaying drug concentration in plasma and whole blood. For drugs with a RBP ≤ 2.0, like FC101a, measuring concentration in plasma rather than whole blood or erythrocytes will increase sensitivity to drug concentration assays (Hinderling 1997). Although it would be advisable to perform assays with whole blood if RBC partitioning were pH dependent, our data predicts S + PrUnbnd (aka f_up_) and S + RBP are pH independent (Hinderling 1997). Therefore *in-vitro* assays should be performed on FC101a with plasma as the assay matrix.

### Potential neurotherapeutic use of FC101a hits

**Fig. 13:**
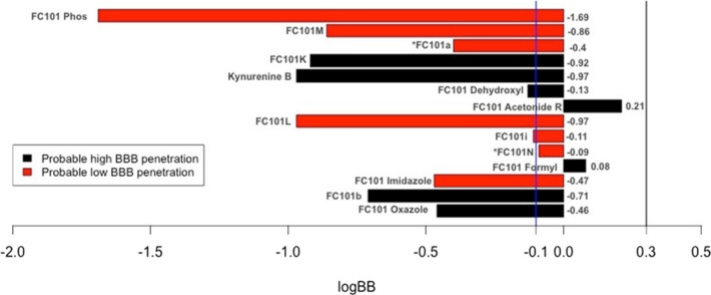
Assessing capability of FC101a and SA to penetrate BBB. Bar chart depicting logBB values of FC101a hits. Color of bars indicate result from a binary “High”/”Low” classifier- red bars indicate “High” probability of penetration and black bars indicate “Low” probability of penetration. The magnitude of each bar indicates the analog’s predicted decimal logarithm of blood–brain partition coefficient. The vertical blue line (−0.1) indicates a regularly reported cut-off between high and low penetration, with higher penetration having more positive values. The vertical black bar indicates the cut-off for values that generally easily penetrate the BBB

FC101a has the potential to be developed as a treatment option for glioblastoma, which are extremely invasive neurological tumors with both a poor clinical response and poor prognosis- median survival is 0.4 years (Mahdavian et al. 2014b; Ohgaki and Kleihues 2005). FC101a has been shown in previous work to induce glioblastoma apoptotic cell death and to significantly reduce the tumor’s migratory capacity (Mahdavian et al. 2014b). Herein lies the bottleneck: in order for FC101a to effectively treat glioblastoma, the drug must efficiently penetrate the BBB. Figure 13 illustrates the likelihood of penetrating the rat BBB; seven analogs are classified as probable high BBB penetrators. Two of the seven, FC101 Formyl (logBB 0.08) and FC101 Acetonide R (log BB 0.21), are the most likely candidates to penetrate the BBB due to their predicted decimal logarithm of blood–brain partition coefficient (logBB), indicated by the magnitude of the bars in Fig. 13. Although the precise cutoff between high and low BBB penetration is unknown and different values have been reported, a log [brain/blood] = −1.0 has been reported regularly, with larger positive numbers indicating higher penetration. This suggests FC101 Acetonide R would be the most likely candidate as a neurotherapeutic agent, although it may require further optimization.

**Fig. 14:**
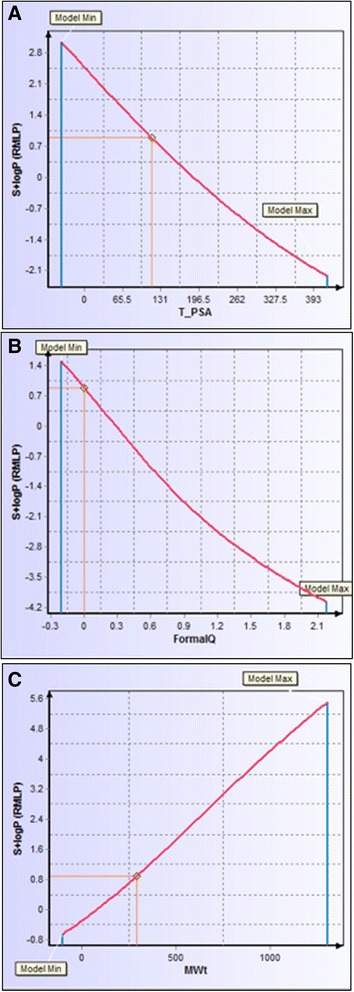
Descriptor sensitivity analysis of logP in response to three most influential descriptors. All three graphs chart the sensitivity of S + logP to a unique descriptor. **a** Response of model to TPSA. Lowering the topological polar surface area of FC101a to approximately 100 would increase S + logP to1.0. **b** Response of model to formal electric charge. FC101a charge is 0, slightly lowering this charge would increase S + logP. **c** Response of model to molecular weight. Increasing MW would raise S + logP

DSA tool found S + logP to be the most influential descriptor to logBB. We suggest structural modifications designed to increase the logP of FC101 Acetonide R will thereby increase logBB; specifically, an increase of logP to 1.0 would result in a logBB of 0.3. Molecules with logBB > 0.3 tend to readily cross the BBB Vilar et al. (2010). We provided hints to structural modifications a medicinal chemist could employ to achieve a higher logP value. The two most influential descriptors of logP are formal electric charge and MW, followed by topological PSA (TPSA). Figure 14 illustrates that lowering formal electric charge and increasing MW, while reducing TPSA, would all contribute to an increase in the logP of FC101a, thus increasing logBB. Increasing MW in order to increase the probability of BBB penetration may seem contradictive; it is generally true that smaller molecules are more likely to penetrate the BBB. Recommendations similar to the RO5 have been proposed regarding molecular parameters contributing to a given molecule’s ability to cross the BBB, i.e., molecules with a molecular mass < 450 Da are more likely to penetrate the BBB (Vilar et al. 2010). Therefore we suggest the medicinal chemist consider the predicted preferred larger MW as a buffer zone.

### Characterization of metabolic and excretion pathways of FC101a hits

**Fig. 15:**
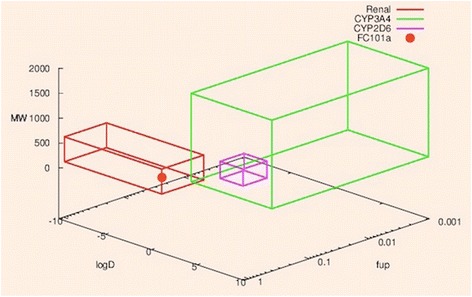
Three-dimensional graph depicting possible FC101a clearance pathway. Three-dimensional graph depicts FC101a clearance pathway (red dot) in relation to boundaries of three clinically significant clearance pathways- pathways were determined by Kusama et al. (2010). Red box indicates cluster of drugs undergoing renal clearance, green box indicates cluster of drugs undergoing CYP3A4 metabolism, and purple box indicates cluster of drugs undergoing CYP2D6 metabolism. Graph was generated at: http://www.bi.cs.titech.ac.jp/CPathPred/pred/rect.html

The role of excretion and metabolism significantly affects the outcome of the drug design process. Understanding these pathways aid in predicting drug-drug interactions (DDI), toxicities, and pharmacokinetics (Hosey et al. 2014). Kusama et al. (2010) grouped 141 approved drugs by charge and plotted log D, MW, and f_up_ in three-dimensional space. Clearance pathways were previously defined for each drug in the dataset, and were determined to be metabolism by CYP3A4, CYP2C9, CYP2D6, hepatic uptake by OATPs, or unchanged renal excretion. Boxes were drawn around each clearance pathway; the mathematical criterion for drawing the boxes is “maximizing F value (harmonic mean of precision, and recall) with minimum volume.” FC101a was plotted as previously described. Figure 15 depicts MW, S + logD, and S + PrUnbnd of FC101a (red dot) within the defined three-dimensional border for unchanged renal excretion. Three-dimensional graph was generated using free software found at: http://www.bi.cs.titech.ac.jp/CPathPred/pred/rect.html, (Kusama et al. 2010). Additional file 3 outlines the prediction accuracy of each model used within this paper, including logD (S + logD) and S + PrUnbnd (f_up_). The external test set consists of 1,793 logD measurements and 102 f_up_ measurements, none of which were included in the respective training sets. Both S + logD and S + PrUnbnd models performed well, with R^2^ value of 0.8907 and 0.68, respectively [See Additional file 3]. All structures fell within the AD of each model. It has not escaped our attention that the previously mentioned classification system only examines three members of the cytochrome P450 family, which accounts for 75 % of total drug metabolism in humans (Guengerich 2008). To scrutinize our current data, which suggests FC101a is stable and may clear un-metabolized via the renal system; our lab is currently conducting further *in-silico* assays and *in-vitro* microsomal assays to determine if FC101a interacts with any member of the cytochrome P450 family.

### Determining potential toxicity of FC101a hits

Animal toxicity issues account for 11 % of total attrition in drug development, and 20-40 % of drug failures are attributed to toxicity concerns (Kennedy 1997). Therefore, we devoted significant attention to determining the potential toxicity of FC101a. Previous mouse xenograft studies have shown FC101a to be well-tolerated and non-toxic at dosages as high as 8 mg/kg/day, which resulted in a 30 % reduction in tumor size (Mahdavian et al. 2014a). Two prediction models, TOX_Rat and TOX_BRM_Mouse, determine LD_50_ for lethal rat acute toxicity to be 1587.27 mg/kg, and TD_50_ for tumor induction in mouse population over a standard lifespan to be 1544.41 mg/kg/day orally, respectively- a near 200 fold increase in the dose required to reduce tumor size by 30 %.

FC101a was subjected to two over-arching toxicity prediction models that rank compounds by accumulating all toxicity related ranking criteria into two columns. TOX_MUT_Risk is a virtual Ames test summarizing 10 mutagenic models that represent expected mutagenicity for five strains of *Salmonella typhimurium*. The second model, TOX_Risk, takes into account TOX_MUT_Risk and six other rules focusing on acute toxicity, cardiac toxicity, carcinogenicity, and hepatotoxicity. Each analog returned a TOX_Risk score of 0, but only three analogs returned a 0 score for both filters- FC101 Oxazole, FC101 Imidazole, and FC101 Phos (see Table 5). FC101 Imidazole and FC101 Phos predicted maximum recommended therapeutic dose (S + MRTD) is < 3.16 mg/kg/day, less than 8 mg/kg/day required to reduce tumor size by 30 % in mouse xenograft models; therefore, leaving FC101 Oxazole as the most promising low toxic SA. From here, we focused on FC101a positive results (see Table 6). Three strains of *S. typhimurium* are potentially sensitive to mutagenic effects of FC101a and/or its liver metabolites. Our lab plans to validate results by performing *in-vitro* Ames Salmonella/microsomal assays with all five strains. TOX_ER_filter predicts FC101a is likely to bind detectably to estrogen receptor in rats with a relative binding affinity of 48 % (100 % * (IC_50_ 17βestradiol / IC_50_ FC101a)). Estrogen receptor affinity is another area requiring *in-vitro* assessment in order to decrease the risk of toxicological late stage failure.

**Table 5:** Overall toxicity predictions for FC101a using TOX_MUT_Risk and TOX_Risk filters

Analog	TOX_MUT_Risk	TOX_Risk
FC101 Oxazole	0	0
FC101b	1	0
FC101 Imidazole	0	0
FC101 Formyl	1	0
FC101N	1	0
FC101i	1	0
FC101L	1	0
FC101 Acetonide R	1	0
FC101 dehyroxyl	1	0
Kynurenine B	1	0
FC101K	1	0
FC101a	1	0
FC101M	1	0
FC101 Phos	0	0

Filters provide a qualitative estimate of potential toxicity concerns. Approximately 16 % of commercial drugs within the focused subset of the WDI receive a TOX_MUT_Risk score > 1 and approximately 4 % have a score > 2. TOX_Risk evaluates overall toxicological concerns and is greater than 3.3 for ~10 % of the focused WDI

**Table 6:** Positive toxicity model prediction results for FC101a

Toxicity model	Output	Model definition
TOX_ER_filter	Toxic (48 %)	Qualitative assessment of estrogen receptor toxicity in rats.
TOX_MUT_97 + 1537	Positive	Qualitative assessment of mutagenicity of the pure compound in TA97 and/or TA1537 strains of *S. typhimurium.*
TOX_MUT_m97 + 1537	Positive	Qualitative assessment of mutagenicity of the compound and its microsomal rat liver metabolites in TA97 and/or TA1537 strains of *S. typhimurium.*
TOX_MUT_m98	Positive	Qualitative assessment of mutagenicity of the compound and its microsomal rat liver metabolites in TA98 strain of *S. typhimurium.*

Of 20 applicable toxicity prediction models, four returned positive results

Transporter protein P-gp can mediate toxic DDIs (Hosey et al. 2014), largely due to its wide substrate spectrum overlap with CYP3A4 (Finch and Pillans 2014). For example, rosuvastatin uptake is inhibited by cyclosporine and results in a sevenfold increase in AUC, potentially leading to serious side effects (Simonson et al. 2004). ADMET Predictor’s S + Pgp_Substrate model was used to predict the likelihood of P-gp efflux. FC101a was classified as a non-substrate with a 97 % likelihood of no P-gp efflux, lowering the perceived risk of P-gp mediated DDIs.

**Fig. 16:**
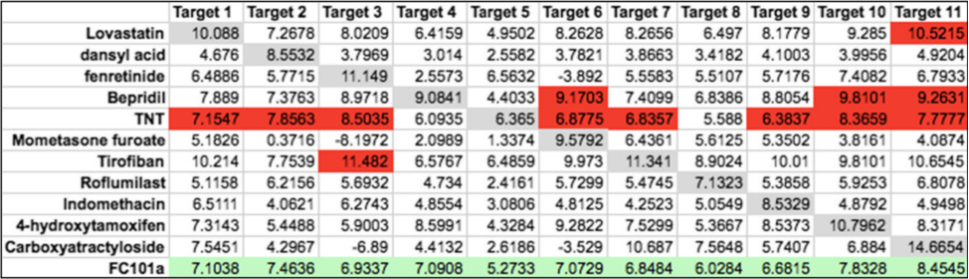
*In-silico* specificity matrix based on eleven randomly chosen FDA approved drugs. Two-dimensional specificity matrix generated *in-silico.* Matrix was generated using binding affinity scores calculated from SYBYL-X Total Score- a robust scoring function. The gray diagonal line indicates binding between drugs and their therapeutic target- high binding scores should only occur along the diagonal line, if drugs are highly specific for their intended therapeutic target. Docking scores with FC101a (green row) do not exceed any scores along the gray line, possibly indicating that FC101a is specific for its unknown target(s). Four of the eleven drugs bind strongly to at least one non-target, indicative of low target specificity (red cells). Drugs 2, 3, 6, and 8–11 are specific to their respective targets

Drugs with low target specificity often bind to multiple sites, thereby increasing the required minimum dose and the probability of unwanted side effects. Figure 16 illustrates a specificity matrix containing docking scores between eleven biological targets, respective FDA approved drugs, and FC101a. High affinity binding should only occur along a diagonal line that represents affinity between each target and its respective drug. For drugs expressing high target specificity, the cells are highlighted in gray; cells highlighted in blue indicate high affinity binding to a non-target, indicating low specificity. FC101a binds weakly to each of the eleven targets, a characteristic of highly specific molecules with low toxicity. A more complete computational strategy for addressing specificity would involve virtually docking FC101a with all potential targets within a cell; however, a complete database for this approach is currently non-existent. Our method of using targets that span all known classes of therapeutic targets is the beginning for others to build such a database (Shaikh et al. 2007). To increase the accuracy of future models, we will choose multiple sets of 11 random targets, each from a class of known therapeutic targets.

### Potential anti-cancer mechanisms of action

**Fig. 17:**
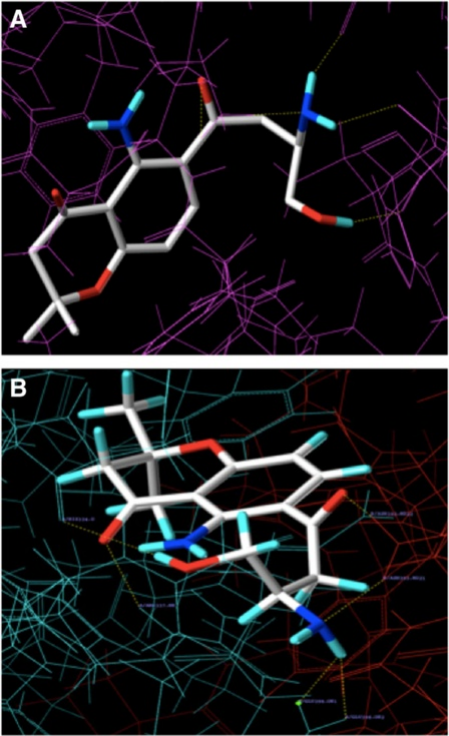
FC101a docked into two highest scoring proteins. **a** Three-dimensional rendering of FC101a bound to binding site in top ranked protein, procaspase-8 (PDB 2K7Z). **b** FC101a clearly interacting with regions from both chain A (green) and chain B (red) of the Caspase-8: cFLIP complex

**Fig. 18:**
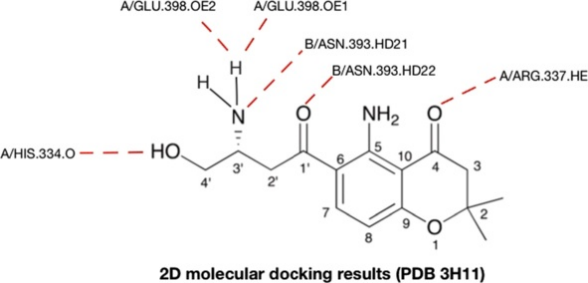
Two-dimensional rendering of FC101a bound to procaspase-8 – cFLIP(L) complex. Dash red lines indicate interactions between FC101a and both proteins from PDB ID 3H11. More specifically FC101a interacts with *i)* oxygen of His334, two oxygen atoms of side chain carboxyl group of GLU398, hydrogen atom of side chain nitrogen from ARG337 of chain A, and ii) two hydrogen atoms of side chain amide nitrogen from ASN393 of chain B

We performed 49 molecular dockings between unambiguous protein structures involved in caspase mediated extrinsic apoptosis and FC101a, FC101b, and kynurenine. Table 7 depicts docking scores with FC101a. PDB ID 2K7Z (Total Score 8.7926), which represents the monomeric unprocessed catalytic domain of the caspase-8 zymogen (procaspase-8); and PDB ID 3H11 (Total Score 8.6323), the crystal structure of the protease-like domain of cellular FLICE- inhibitory protein (cFLIP(L)) in complex with procaspase-8 are the top two ranked proteins with the highest affinity for FC101a are (see Fig. 17). We propose a mechanism of action by which binding of FC101a to either or perhaps both the protease-like domain of cFLIP(L) and procaspase-8, as shown in Fig. 17, could enhance heterodimerization between the two molecules, thus enhancing caspase activity and leading to apoptosis upregulation. Our proposed mechanisms are based on four observations; substantial levels of cFLIP have been recorded in human cancers such as ovarian, colon, glioblastoma, breast, colorectal, and prostate, and it has been recognized as a major regulator of apoptosis, utilizing dual functionality- cFLIP(L) inhibits caspase-8 activation at high expression levels, but increases caspase-8 activity at low levels (Safa et al. 2008; Bagnoli et al. 2010); prior to caspase-induced apoptosis induction, procaspase-8 must be recruited to FADD and form the DISC, followed by caspase heterodimerization and proteolytic cleavage; cFLIP(L) is capable of increasing the proteolytic activity of caspase-8 by facilitating heterodimerization of the two molecules, yet the mechanism forcing these two molecules together and thereby leading to enhanced initiator caspase activation remains unclear (Yu et al. 2009); and finally, FC101a interacts with regions from both chain A and chain B of the caspase-8: cFLIP complex, illustrated in Fig. 18, and docking with either chain independent of the other resulted in scores below the minimum allowed threshold. Our logic is in-line with the notion proposed by others that drugs targeting caspase-8 isoforms and c-FLIP variants may offer a therapeutic advantage (Safa et al. 2008). Nonetheless, *in-vitro* analysis must be performed in future studies to confirm or eliminate our proposed mechanisms.

**Table 7:** Molecular docking affinity scores between FC101a and potential biological targets

PDB ID	Total score	C score
2K7Z	8.7926	5
3H11	8.6323	5
3M0D	7.2915	5
4AUQ	7.1868	5
2Y1L	6.677	5
1IBX	6.4214	5
1IBX*	6.0164	5
1G5J	5.9451	5
3MQP	5.7313	5
3M0A	5.3557	5
2ROC	3.9611	5
4JR2	7.7731	4
3ZLN	7.7672	4
4MSV	7.7402	4
4JB8	7.2884	4
3WIX	7.1413	4
3EZQ	6.9801	4
2BID	6.3079	4
4BD2	6.2248	4
3 M06	5.9925	4
3QO4	5.9596	4
3YGS	5.676	4
3M0D**	5.1542	4

Proteins are ranked in descending order. *Chain B was removed during preparation. **Chains A, B, and D were removed during preparation

## Conclusion

FC101a demonstrates tremendous potency towards multiple cancer cell lines, yet exhibits modest *in-vivo* anti-cancer activity. Potential therapeutic compounds are useless without having a desirable PK profile, and thus it is vital to find the source of such diminished potency if one wishes to develop a marketable drug. In addition to investigating the novel mode of action of FC101a, this study was designed, in-part, to answer two questions utilizing primarily *in-silico* techniques: *why is in-vivo potency diminished*, and *how can we improve in-vivo potency?* Objectively, we aimed to characterize the ADMET profile of FC101a, to determine those structural analogs with the most promising ADMET profile, to suggest methods of PK optimization, and to elucidate potential biological targets of FC101a.

We verified FC101a compliance  with both Lipinski’s RO5 for orally bioavailable drugs and with Veber’s selective criteria for rat oral bioavailability > 20-40 %. We analyzed each ADMET component of FC101a to identify molecular properties and descriptors contributing to a successful PK profile, and properties requiring optimization. The most relevant computed ADMET descriptors and values are displayed  in Table 8 for both FC101a and those structural analogs selected for synthesis and anti-cancer potency assessment. Additionally, FC101 Phos was included for comparison, given its least desirable performance. Recommended range of values for *i)* 95 % of orally bioavailable drugs or *ii)* conservative threshold to risk determined by ADMET Predictor Version 7.1 were included for each value. We accumulated more evidence suggesting FC101a possesses high potential for drug development. Furthermore, we identified FC101 Oxazole (preferred due to lower predicted MRTD) and FC101 Imidazole as the most optimized analogs, and given their higher lipophilic logD, it is possible potency could be increased thereby addressing FC101a’s modest *in-vivo* activity. We were unable to eliminate any compound from our group of analogs based S + Absn_Risk and S + ADMET_Risk scores, although FC101 Phos performed the worst. We found FC101a to have low risk scores from four physiochemical and biopharmaceutical filters, compliance with the RO5 and Veber’s druglike indices, desirable predicted aqueous solubility (logS, S + Sw, Fsp^3^, and hydrophobic logD), logP, effective and apparent permeation values, possible un-metabolized clearance via the renal system, high target specificity, and no affinity for P-gp. Additionally we identified FC101 Formyl and FC101 Acetonide R as the analogs most likely to penetrate the BBB. Secondly, and perhaps most important, we identified areas of concern. These areas of concern require further *in-vitro* testing and include:

- FC101a potential mutagenic affects on *S. typhimurium* strains TA97, TA1537, and TA98.
- FC101a potential estrogen receptor toxicity, although the ratio binding percent was relatively low.

**Table 8:** Important computed ADMET properties and their recommended ranges for orally active drugs

Property	Description	Recommended range	FC101a	FC101 Oxazole	FC101 Imidazole	FC101 Phos
MW	Molecular weight in Da	<450	292.34	300.32	299.33	372.32
PSA	Surface sum of all polar atoms and attached atoms in Å^2^	<140	110.708	89.708	94.106	**167.62**
Nrot	Number of rotatable bonds	<10	4	3	3	6
HBD	Number of hydrogen bond donors	<5	3^a^	1	2	4
HBA	Number of hydrogen bond acceptors	≤8	6	6	6	**9**
LogP	Octanol-water partition coefficient	≤4.5	0.89	2.35	2.14	−0.91
LogD	Octanol-water distribution coefficient	≤3.5	−0.53	2.35	2.11	−0.9
Sw	Aqueous solubility in mg/ml	≥0.010	2.34	0.0261	0.0259	0.7740
LogS	Log of aqueous solubility	−6.0 – 0.5	−3.39	−4.29	−3.66	−1.81
MDCK	Apparent Madin-Darby canine kidney cell permeability in cm/s × 10^7^	≥30	44.79^a^	313.79	212.86	**14.82**
Peff	Human effective jejunal permeation in cm/s × 10^4^	≥0.5	0.75^a^	3.29	2.36	**0.29**
Fsp^3^	Fraction of sp3 C to total C atoms	>0.36	0.467	**0.313**	**0.313**	0.467
RBP	Blood-to-plasma concentration ratio	<1.0	0.97^a^	0.81	0.85	0.62
Vd	Volume of distribution in L/kg	≤3.7	**6.7**	2.76	**3.82**	0.6
F_up_	Percent of drug unbound to plasma proteins	>10 %	66.61	13.1	15.35	36.48
MolVol	Molal volume at normal boiling point in cm^3^/mol	<475	308	294	301	364
hERG	pIC50 as measure of affinity for hERG K+ channel	≤5.5	4.51	4.24	4.53	3.76
MRTD	Maximum recommended therapeutic dose in mg/kg/day	>3.16	>3.16	>3.16	**<3.16**	**<3.16**
TOX	Risk summary for mutagenic potential in *S. typhimurium*	≤1	1^a^	0	0	0
MUT						
Risk						
Absn_Risk	Risk summary for oral absorption	<3.5	0.99	0	0	**3.5**
ADMET_Risk	Summary of all predicted ADMET risk factors	≤6.5	1.99	0	0.06	3.5

Recommended ranges are determined by range of 95 % of orally bioactive drugs and/or cut-offs pre-determined by ADMET Predictor Version 2.0, as defined below. Seven of the cut-offs are within the range of 95 % of orally bioactive drugs. Molar weight (range for 95 % of drugs: 130–725 Da); LogP (range for 95 % of drugs: −2 to 6.5); HBA (range for 95 % of drugs: 2–20); HBD (range for 95 % of drugs: 0–6); nrot (range for 95 % of drugs: 0–15); MDCK permeability in nm/s (range for 95 % of drugs: < 5 low, > 500 high); LogS (range for 95 % of drugs: −6.0 to 0.5) (Ntie-Kang et al. 2013); TOX_MUT_Risk, Absn_Risk, ADMET_Risk, Sw, Peff, MDCK, MW, Mol Vol, LogD, Vd, F_up_, and hERG pIC_50_ exact cut-offs pre-determined by ADMET Predictor. (ADMET Predictor V2 Manual); PSA (<140 based on Veber et al. (2002)); Fsp^3^ range based on Lovering et al. (2009). Bolded values indicate values outside the range of 95 % of orally bioactive drugs and/or exceeding risk cut-off. ^a^Values indicate properties likely needing optimization or *in-vitro* assessment

Areas of likely needed optimization include:

- Hydrogen bond donor charge
- Apparent and effective permeability
- Blood-to-plasma concentration ratio
- Volume of distribution

Our third objective was to discover potential avenues medicinal chemists could use to optimize FC101a; therefore, we analyzed each ADMET component of FC101a to identify properties with the potential for modification. Subsequently, we hinted at avenues by which a medicinal chemist could optimize effective permeation, BBB penetration, and aqueous solubility. The means of accomplishing these changes is beyond the scope of this paper, and is of course an oversimplification. Structure based differences play a major role in oral bioavailability and it is not surprising that difficulties are encountered during the optimization of drugs. Nonetheless, our data provides medicinal chemists hints for structural modifications that could achieve more desirable PK features. Diminished *in-vivo* activity can now be addressed and scientists can begin to further understand the mechanisms behind such behavior. *In-silico* data should be followed up with *in-vitro* assays, and currently our lab is in the process of doing so.

In order to investigate the mechanism of action for FC101a, our fourth objective, we designed a docking protocol to elucidate possible biological targets. A consensus scoring function and protocol was designed by docking hKAT II with its known biological substrate, kynurenine – a naturally occurring metabolite sharing structural similarities with FC101a. The protocol was applied to 49 proteins involved in caspase-mediated apoptosis. Docking results led to proposing three similar mechanisms of action by which FC101a facilitates heterodimerization of procaspase-8 by targeting either or both procaspase-8 and cFLIP(L), strengthening the affinity between the two molecules and thus enhancing extrinsic apoptosis. Investigating these potential targets warrant further *in-vitro* assessment.

Future FC101a research efforts should focus on discovery of new potential targets involved in apoptosis promoting pathways, assessment of proposed biological target(s), *in-vitro* validation of *in-silico* results presented within (i.e., Ames test, microsomal assays, drug concentration in plasma), and enhancing *in-vivo* potency via PK optimization. Additionally, researchers interested in developing a potentially potent treatment for glioblastoma tumors should focus on optimizing FC101 Formyl and FC101 Acetonide R to penetrate the BBB; although, these analogs should first be assessed for anti-cancer activity. In conclusion, FC101a’s high specificity and promising ADMET profile, along with its documented potent anti-cancer activity, render this molecule a promising lead candidate for a low toxic anti-cancer agent effective against a broad range of cancers. FC101a should continue to be developed and prepared for translational drug studies; first generation structural analogs, FC101 Oxazole and FC101 Imidazole, should be explored as lead candidates.

## Additional files

Additional file 1:
**ADMET Predictor clean output file.** Contains all predicted descriptors, parameter settings, and out-of-scope indicator column. Additional file 2:
**Original 2D SDF file used to generate ADMET values.** The file contains more than 14 structures, despite only 14 being covered in this original manuscript. Some structures are enantiomers, which are unrecognizable by ADMET Predictor Version 7.1. Additional file 3:
**Statistical measurement of binary and linear predictive models in order to estimate prediction accuracy of ADMET Predictor.** Contains concordance, sensitivity, and specificity of all binary models used in this study; contains R^2^ values and n for both training and test sets for all models used in this study. Additional file 4:
**Detailed account of molecular docking protocol for caspase-related proteins.** Detailed account of the steps, protocol, and settings used to dock leadset4 with all 49 proteins of interest. Details are of most significance to the SYBYL-X user. Additional file 5:
**Specificity matrix design and SYBYL-X molecular docking detailed protocol.** Detailed experimental section for the retrieval, preparation, and molecular docking of 11 FDA approved drugs with their therapeutic target. Data used to design specificity matrix and to investigate FC101a’s specificity. Details are of the most significance to the SYBYL-X user. Additional file 6:
**Chemical structure of FC101a structural analogs.** Chemical structure and short description of the altered functionality of each compound.

## Abbreviations

FC101a: Fusarochromanone- parent compound
PK: Pharmacokinetic
ADMET: Absorption, distribution, metabolism, excretion, toxicity
BBB: Blood brain barrier
PARP: Poly (ADP-ribose) polymerase
SA: Structural analog(s)
S+: Simulations-plus symbol that indicates the following value is a predicted value using their (Simulations-Plus) proprietary software
ANNE: Artificial neural network ensemble is a collection of ANNs whose outputs are averaged to minimize errors from each individual ANN
ANN: Artificial neural network is a connected set of equations with coefficients that are optimized to minimize the error between predicted and observed values for an output. The network consists of inputs, neurons, and an output
BA: Bioavailability
S+ logD: Octanol-water distribution coefficient
S+ Sw: Aqueous water solubility in mg/ml
S+ P_eff_-: Effective permeability in the human jejunal
S+ MDCK: Apparent Madin Darby Canine Kidney COS permeability
S+ PrUnbnd: Percent of drug unbound to plasma proteins, aka F_up_
S+ RBP: Blood-to-Plasma concentration ratio
S+ logBB: Logarithm of the blood–brain partition coefficient
MW: Molecular weight
P-gp: Permeability-Glycoprotein
DSA: Descriptor sensitivity analysis tool by ADMET Predictor
AD: Applicability domain refers to the region of molecular descriptor space occupied by all the drugs used to train a prediction model
hKAT-II: Human Kynurenine Aminotransferase II
Threshold: One of the two parameters determining the extent of the protomol. This factor (between 0.01 and 0.99) indicates how much the protomol can be buried in the protein. The default of 0.50 is adequate in most circumstances. Increasing this number will decrease the volume
Bloat: This second parameter provides a way to inflate the protomol. Use the slider to specify the number of Å (0–10) used to expand the protomol in 3D. Changing this parameter may affect the protomol’s volume in the following situations: 1) With a longer distance, the protomol can reach into crevices. 2) If the active site is a channel, rather than a cavity, longer distances may be required to better define the protomol at the open ends of the channel
MCS mode: Multi-Channel Surface mode
Total Score: An empirically derived scoring function that is based on the binding affinities of protein-ligand complexes and on their X-ray structures. Expressed as –log(k_d_)
C Score: Consensus scoring function. It is the simultaneous use of multiple different scoring functions in order to make more accurate predictions
nrot: Rotatable bond count
HBD: Hydrogen bond donor
HBA: Hydrogen bond acceptor
PSA: Polar surface area in angstroms squared
S+ logP: Octanol-water partition coefficient
VD: Volume of distribution
RBC: Red blood cell partitioning
TPSA: Topological Polar Surface Area in Angstroms squared
DI: Drug-drug interaction(s)
UC: Area under curve, aka integral, is plot of drug concentration in blood plasma against time
ADD: Fas-Associated protein with death domain
ISC: Death-inducing signaling complex
