# Supplementary material for: Use of in-silico assays to characterize the ADMET profile and identify potential therapeutic targets of fusarochromanone, a novel anti-cancer agent
Source: In Silico Pharmacol. 2015 Jun 4;3:6. doi: 10.1186/s40203-015-0010-5 (PMC4464579; doi:10.1186/s40203-015-0010-5)
Supplement: Additional file 6: — Chemical structure of FC101a structural analogs. Chemical structure and short description of the altered functionality of each compound. [file 40203_2015_10_MOESM6_ESM.pdf]

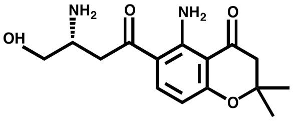

FC101-Parent

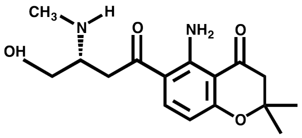

1

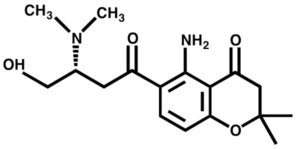

2

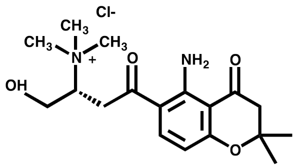

3

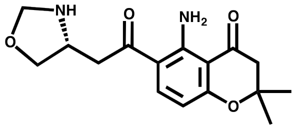

4

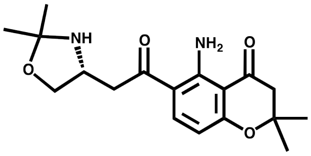

5

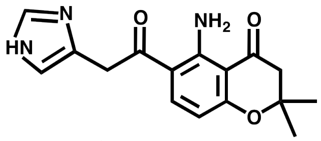

6

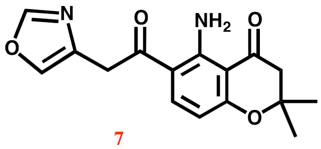

7

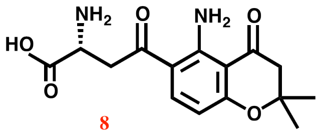

8

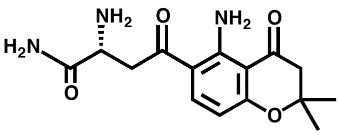

9

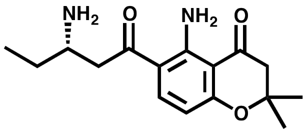

10

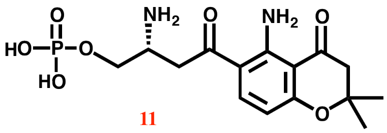

11

1: Methylated nitrogen: FC101N

2: Di-methylated nitrogen: FC101i

3: Tri-methylated nitrogen: FC101L

4: Formyl functional group: FC101 Formyl

5: Acetonide functional group. Saved as: FC101 Acetonide R

6: Imidazole functional group: FC101 Imidazole

7: Oxazole functional group: FC101 Oxazole

8: Additional beto-ketone functionality: FC101M

9: Additional bet-ketone and amine functionality: FC101K

10: Removed hydroxyl: FC101 Dehyroxyl

11: Phosphorylated. Saved as: FC101 Phos
